# Molecular identification of *Theileria* parasites of northwestern Chinese Cervidae

**DOI:** 10.1186/1756-3305-7-225

**Published:** 2014-05-14

**Authors:** Youquan Li, Ze Chen, Zhijie Liu, Junlong Liu, Jifei Yang, Qian Li, Yaqiong Li, Shuangqing Cen, Guiquan Guan, Qiaoyun Ren, Jianxun Luo, Hong Yin

**Affiliations:** 1 State Key Laboratory of Veterinary Etiological Biology, Xujiaping 1, Lanzhou, Gansu 730046, PR China; 2 Key Laboratory of Veterinary Parasitology of Gansu Province, Xujiaping 1, Lanzhou, Gansu 730046, PR China; 3 Lanzhou Veterinary Research Institute, Chinese Academy of Agricultural Science, Xujiaping 1, Lanzhou, Gansu 730046, PR China

**Keywords:** *Theileria* and *Babesia*, Molecular detection, Cervid, PCR, Northwestern China

## Abstract

**Background:**

*Theileria* and *Babesia* protozoan parasites are transmitted mainly by tick vectors. These parasites cause heavy economic losses to the live-stock industry, as well as affecting the health of wild animals in parasite-endemic areas. Identification of infectious agents in wild animals is not only crucial for species preservation, but also provides valuable information on parasite epidemiology. Here, we conducted a molecular surveillance study in Northwestern China to assess the prevalence of blood pathogens in cervids.

**Methods:**

PCR analysis and microscopic evaluation of blood smears to detect *Theileria*- and *Babesia*-related diseases in Cervidae were conducted, in which 22 blood samples from red deer (n = 22) in Qilian Mountain and 20 from sika deer (n = 20) in Long Mountain were collected and tested for the presence of *Theileria* and *Babesia*. The 18S rRNA gene was amplified, and selected polymerase chain reaction (PCR)-positive samples were sequenced for species identification.

**Results:**

PCR revealed that 9.1% of the Qilian Mountain samples and 20% of the Long Mountain samples were positive for *Theileria uilenbergi*; 90.09% of the Qilian Mountain samples (n = 22) were positive for *T. capreoli*, but all of the Long Mountain samples (n = 20) were negative for *T. capreoli*; no other *Theileria* or *Babesia* species were found. PCR showed that *T. uilenbergi* and *T. capreoli* were present in red deer in Qilian Mountain, while only *T. uilenbergi* was found in sika Deer in Long Mountain. The 18S rRNA gene sequences were aligned against the corresponding GenBank sequences of known isolates of *Theileria* and *Babesia* and subjected to phylogenetic analysis. The phylogenetic tree showed that the newly isolated *Theileria* spp. could be classified as belonging to two clades: one group belonged to the same clade as *T. uilenbergi*, the other to a clade containing *T. capreoli*.

**Conclusions:**

Our results provide important data to increase understanding of the epidemiology of Cervidae theileriosis, and will assist with the implementation of measures to control theileriosis transmission to Cervidae and small ruminants in central China.

## Background

*Theileria* and *Babesia*, parasites that are mainly transmitted by tick vectors, cause heavy economic losses to live-stock and affect the health of wild animals where ever such parasites are endemic. *Theileria* and the closely related genus, *Babesia*, exhibit complex life cycles that involve mammalian intermediate hosts and hard ticks as the definite hosts [1]. *Theileria* is an obligatory intracellular parasite that infects leukocytes and erythrocytes in the intermediate host, unlike *Babesia*, which only infects erythrocytes. Among the *Theileria* and *Babesia* parasites that infect cervids, several *Theileria* spp. (e.g. *T. cervi*, *T. capreoli*, *Theileria* sp. OT3, *Theileria* sp. ZS OT4, and *T. ovis*), and *Babesia* spp. (e.g. *B. bigemina*, *B. bovis*, *B. capreoli*, *B. divergens*, and *B. odocoilei*) are described as being moderately pathogenic or benign. *Theileria* and *Babesia* are cosmopolitan parasites [2] that have been detected in wild ruminants in many countries including Japan [3-5], South Korea [6], Brazil [7,8], the United States [9-12], Italy [13], eastern Austria [14], northern and central Spain [15-17], and the United Kingdom [18]. In China, He *et al*. [19] first reported the existence of *Theileria* infection in sika deer in the Hubei province of central China.

Although there are no published epidemiological data on the Cervidae of northwestern China, no suspected cases of theileriosis in small ruminants have been observed here. In the current study, we performed an epidemiologic survey of *Theileria* and *Babesia* infections of Cervidae in northwestern China. Polymerase chain reaction (PCR) analysis and microscopic examination of blood smears were used for species identification and to elucidate the evolutionary relationships among the newly identified hemoparasite species based on the 18S rRNA gene sequences of known *Theileria* and *Babesia* species.

## Methods

### Sample collection

The two regions investigated in northwestern China are located at latitudes 34° 44′ to 73° 28′ north and longitudes 97° 20′ to 106° 35′ east. The study period was August 2013. Twenty-two blood samples (n = 22) were collected randomly from wild red deer in Qilian Mountain, and 20 blood samples (n = 20) were collected randomly from domesticated sika deer at a ranch in Long Mountain, northwestern China. Blood smears were prepared for both groups (Table 1). During the blood collection process, cases of suspected theileriosis and babesiosis were investigated. Theileriosis and/or babesiosis should be suspected in tick–infested animals with a fever, enlarged lymph nodes (only for theileriosis), anemia and jaundice, or hemoglobinuria (only for babesiosis).

**Table 1:** Sequences of the oligonucleotide primers used in this study

**Pathogen**	**Target gene**	**Oligonucleotide sequences (5′-3′)**		**Final amplicon size (bp)**	**Reference**
		**Forward**	**Reverse**		
Hemoparasite	18S rRNA	AACCTGGTTGATCCTGCCAGT	GATCCTTCTGCAGGTTCACCTAC	1750	Medlin et al., [20]
*Theileria* spp.	18S rRNA	AGTTTCTGACCTATCAG	TTGCCTTAAACTTCCTTG	1098	Allosop et al., [21]
*T. annulata*	30 kDa	GTAACCTTTAAAAACGT	GTTACGAACATGGGTTT	721	d’Oliveria et al., [22]
*T. sergenti*	MPSP	CACGCTATGTTGTCCAAGAG	TGTGAGACTCAATGCGCCTA	875	Liu et al., [23]
*T. sinensis*	MPSP	CACTGCTATGTTGTCCAAGAGATATT	AATGCGCCTAAAGATAGTAGAAAAC	887	Liu et al., [23]
*T. orientalis*	MPSP	CTTTGCCTAGGATACTTCCT	ACGGCAAGTGGTGAGAACT	776	Ota et al., [24]
*T. cervi* (Type F)	18S rRNA	TTCCCTTTGAGGGGT	AAGCCTATTCCCGTACC	654	Boes et al., [25]
*T. cervi* (Type G)	18S rRNA	CTTCCCGTTATGGAGG	CTTAACCTATTCCCGTGAG	654	Boes et al., [25]
*T. luwenshuni*	18S rRNA	GGTAGGGTATTGGCCTACTGA	TCATCCGGATAATACAAGT	340	Yin et al., [26]
*T. uilenbergi*	18S rRNA	GGTAGGGTATTGGCCTACCGG	ACACTCGGA AAATGCAAGCA	340	Yin et al., [26]
*T. ovis*	18S rRNA	TCGA-GACCTTCGGGT	TCCGGACATTGTAAAACAAA	520	Altay et al., [27]
*T. lestoquardi*	18S rRNA	GTGCCGCAAGTGAGTCA	GGACTGATGAGAAGACGATGAG	785	Aktas et al., [28]
*T. capreoli*	18S rRNA	gtcttgtaattggaatgatgg	ccaaagactttgatttctctc	580	Slemenda et al., [29]
*Babesia* spp.	18S rRNA	TGTCTTGAATACTT(C/G)AGCATGGAA	CGACTTCTCCTTTAAGTGATAAC	950	Ramos et al., [10]
*B. odocoilei*	18S rRNA	CACCGTATTTTGACTTTTTGTCGACTGTCGG	CCCGTAACGGACGAACCTTCTCACGGG	900	Ramos et al., [10]
*B. bovis*	18S rRNA	CTGTCGTACCGTTGGTTGAC	CGCACGGACGGAGACCGA	541	Guido et al., [30]
*B. ovata*	AMA-1	GATACGAGGCTGTCGGTAGC	AGTATAGGTGAGCATCAGTG	504	Sivakumar et al., [31]
*B. bigemina*	18S rRNA	TGGCGGCGTTTATTAGTTCG	CCACGCTTGAAGCACAGGA	1124	Guido et al., [30]
*B. ovis*	18S rRNA	TGGGCAGGACCTTGGTTCTTCT	CCGCGTAGCGCCGGCTAAATA	549	Aktas et al., [32]
*B. motasi*	18S rRNA	AAGAATTTCACCTCTGACAG	GCTTGCTTTTTGTTACTTTG	179	Shayan et al., [33]

### Microscopy of blood smears

Blood smears were air-dried, fixed in methanol, stained with a 10% solution of Giemsa in phosphate-buffered saline (pH 7.2), and then subjected to microscopic analysis and photography.

### DNA extraction

Genomic DNA from 42 whole blood samples was extracted using a genomic DNA extraction kit (Qiagen, Hilden, Germany) according to the manufacturer’s instructions. The DNA yields were determined using a NanoDrop ND-2000 Spectrophotometer (Nanodrop Technologies, Wilmington, DE, USA).

### Molecular detection of *Theileria* and *Babesia* using species-specific primers

PCR was used to detect and differentiate *Theileria* and *Babesia* using species-specific primers [10,20-32], the details of which are shown in Table 2. PCR reactions were performed in an automatic DNA thermocycler (Bio-Rad, Hercules, CA, USA) and PCR products were separated by 1.5% agarose gel electrophoresis to assess the presence of specific bands indicative of *Theileria* spp. and *Babesia* spp.

**Table 2:** **Prevalence of *Theileria *** **and *Babesia *** **in red deer and sika deer in northwestern China, 2013**

**Collection sites**	**Host**	**Prevalence of *Theileria *** **spp. and *Babesia *** **spp. in blood samples**			
		ME^*^	***T. capreoli***	***T. uilenbergi***	***Babesia *** **spp.**
Qilian Mountain	Red deer	72.7% (16/22)	90.9% (20/22)	9.1% (2/22)	0% (0/22)
Long Mountain	Sika deer	10% (2/20)	0% (0/20)	20% (4/20)	0% (0/20)

*ME, microscopic examination of blood smears.

### PCR amplification of the 18S rRNA gene of *Theileria*

PCR was used to amplify protozoan 18S rRNA gene sequences with primers A/B as described by Medlin *et al*. [20]. The DNA fragments generated were ligated into pGEM T easy vectors (Invitrogen, Carlsbad, CA, USA) and transformed into competent JM109 cells (Takara Bio Inc., Shiga, Japan). The transformed bacteria were plated onto selective LB medium, and at least three positive clones identified by the 989/990 primers specific to *Theileria* spp. [21] were sequenced by the GenScript Corporation (Piscataway, NJ, USA). Representative sequences of the newly identified *Theileria* and *Babesia* 18S rRNA genes from this study were deposited in the GenBank database of the National Center for Biotechnology Information (NCBI) (http://www.ncbi.nlm.nih.gov/genbank/).

### Sequence alignments and phylogenetic analysis

**Figure 1:**
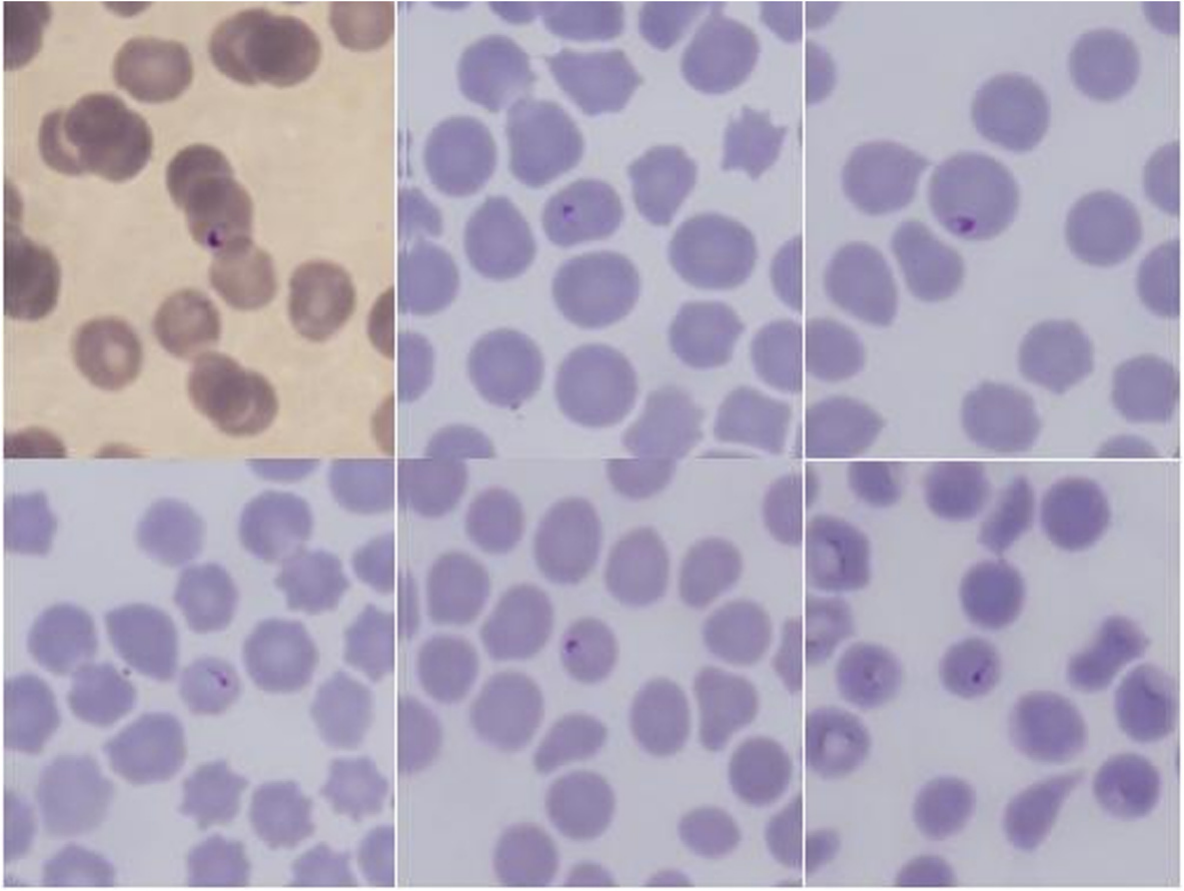
Piroplasms in blood smears from Cervidae.

Compilation, editing, and assembly of the multiple sequences generated from each template were performed using the EditSeq and SeqMan algorithms of the Lasergene software package for Windows (DNASTAR, Madison, WI, USA). Sequence alignment and phylogenetic analysis performed by the MegAlign component of the Lasergene program ver. 4.01 (DNASTAR) were used to perform multiple sequence alignments with the ClustalW algorithm and phylogenetic analysis by the neighbor-joining method. A phylogenetic tree was constructed (Figure 1) based on the *Theileria* and *Babesia* 18S RNA gene sequences determined in the present study, other sequences from our laboratory, and those obtained from GenBank under the following accession numbers: KJ188207-KJ188232, AY726011, DQ866842, AY421708, AY661512, AY661515, EU274472, EU277003, AY260171, AY260172, FJ603460, EU083800, FJ426369, M64243, AY262116, AY262118, AY262119, JX469515, AY262120, JF719835, FJ595120, FJ599640, AB012201, AB012194, HQ188406, AF529272, Z15105, AY260176, AY260177, AY260178, AY260179, AY260180, AB576641, AB012198, GQ304524, AY260180, AY081192, HQ264111, and HQ264112. In the phylogenetic tree, the length of each branch pair represents the distance between sequence pairs, while the units on the horizontal axis indicate the number of substitution events (Figure 1).

### Ethical approval and consent

This molecular approach was then employed to identify *Theileria* parasites of northwestern Chinese cervidae at Lanzhou Veterinary Research Institute, Chinese Academy of Agricultural Science. Use of these clinical samples was approved by the National Review Board (China).

## Results

### Microscopic examination of thin blood smears

Piroplasm infections in the blood smears were observed microscopically in samples from Qilian Mountain (72.7%, 16/22) and Long Mountain (10%, 2/20); all infections exhibited low parasitemia levels (0.01–2.5%) (Table 2). The piroplasms were polymorphous, and most presented as rod-like, needle-like, pear-shaped, or spherical forms (Figure 1). Cases of theileriosis and babesiosis were not seen in the Qilian Mountain and Long Mountain regions of northwestern China.

### PCR detection of *Theileria* and *Babesia* using species-specific primer sets

All 42 blood samples were negative for *Babesia* spp. using *Babesia* genus and *Babesia* species-specific primer sets; 24 of them (n = 42) were positive for *Theileria* spp. using *Theileria* genus and *Theileria* species-specific primer sets. PCR using twenty sets of primers revealed that only two *Theileria* species, *T. capreoli* and *T. uilenbergi*, were detectable in the blood samples. The PCR-positive rates for *T. uilenbergi* in the blood samples from red deer in Qilian Mountain and sika deer in Long Mountain were 9.1% (2/22), and 20% (4/20), respectively. While the PCR-positive rates for *T. capreoli* in blood samples from red deer in Qilian Mountain were 90.9% (20/22), *T. capreoli* was not detected in the blood samples from sika deer in Long Mountain (Table 2). These results confirm that all of the samples that were blood smear-positive were also PCR-positive for *Theileria* parasites. Of the 24 samples for which no piroplasms were observed by microscopy, we found that one was *T. capreoli-* and *T. uilenbergi-positive*, three were *T. capreoli*-positive, and two were *T. uilenbergi*-positive by PCR. Compared with microscopy of blood smears, the PCR method had higher sensitivity and was recommended to be utilized in the diagnosis of piroplasmosis.

### 18S rRNA amplification

The 18S rRNA gene sequences were determined for the *Theileria* spp. isolates from Qilian Mountain and Long Mountain. The length of the 18S rDNA gene was 1,751 bp (*T. capreoli*) and 1,745 bp (*T. uilenbergi*) using the A/B primers, which are specific for hemoparasites. The 18S rRNA gene sequences obtained from red deer and sika deer in northwestern China have been deposited in GenBank under the following accession numbers: KJ188207-KJ188220 for the *T. capreoli* isolates from Qilian Mountain red deer; KJ188227-KJ188232 for the *T. uilenbergi* isolates from Qilian Mountain red deer; and, KJ188221-KJ188226 for *T. uilenbergi* isolates from Long Mountain sika deer.

### Sequence alignments and phylogenetic analysis

**Figure 2:**
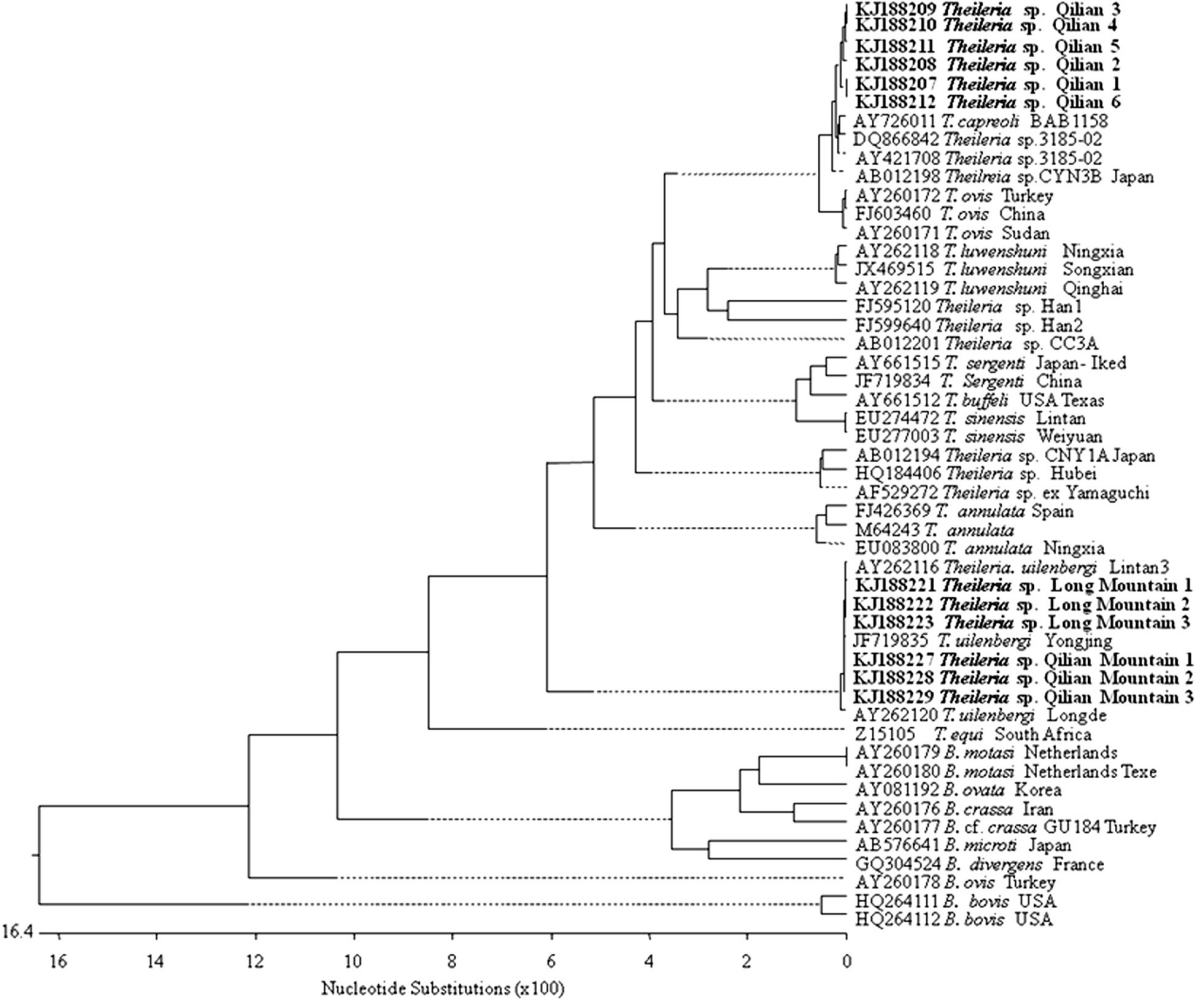
**Phylogenetic tree of *Theileria *** **and *Babesia *** **spp. based on 18S rRNA gene sequences.**

The Basic Local Alignment Search Tool (BLAST) at NCBI was used to analyze the sequences obtained in our study. The results showed that there were two *Theileria* sequences from Cervidae in Qilian Mountain; one shared 99–100% homology with *Theileria* sp. 385/02, *T. capreoli* isolate BAB1158, and *Theileria* sp. CNY3B, while another shared 100% homology with the following isolates: *T. uilenbergi* Longde, *T. uilenbergi* Yongjing, and *T. uilenbergi* Zhangchuan. However, only one species of *Theileria* was found in Long Mountain Cervidae, and its 18S rRNA gene sequence shared 100% homology with the *T. uilenbergi* Longde isolate, the *T. uilenbergi* Yongjing isolate, and the *T. uilenbergi* Zhangchuan isolate. Sequence alignments showed that the newly identified *Theileria* spp. sequences with accession numbers KJ188207-KJ188220 were highly homologous to each other with identity values of 100%, while the other set of newly identified *Theileria* sequences (KJ188221-KJ188232) also were highly homologous to each other with identity values of 100%. Consistent with the BLAST results, the phylogenetic tree constructed in this study showed that one newly sequenced *Theileria* isolate appeared in the same clade as *T. capreoli*, while another appeared in the same clade as *T. uilenbergi* (Figure 2); both of the new 18S rDNA gene sequences show obvious differences to those from *Babesia* (such as those from *B. ovis*, *B. bovis*, *B. motasi, B. ovata*, and *B. divergens*) or other *Theileria* sequences (such as those from *T. ovis*, *T. luwenshunli*, *T. sinensis*, *T. buffei/T. sergenti*, *T. equi*, and *T. annulata*).

## Discussion

To the best of our knowledge, this study is the first to report on cervine theileriosis in northwestern China. Molecular screening for *Theileria* and *Babesia* in red deer and sika deer in northwestern China showed that the most prevalent species of *Theileria* in the deer were *T. capreoli* and *T. uilernbergi*; however, no *Babesia* parasites were found. The prevalence of *T. capreoli* was very high in Qilian Mountain deer, unlike *T. uilenbergi*, the prevalence of which was relatively low in Qilian Mountain. In contrast, the prevalence of *T. uilenbergi* was relatively low in Long Mountain, and no *T. capreoli* infections were found at this location. In both of the areas investigated, no *Babesia* spp. (including *B. odocoilei*, *B. bovis*, *B. ovata*, *B. bigemina*, *B. ovis*, and *B. motasi*) or infections with other *Theileria* spp. (including *T. cervi*, *T. luwenshuni*, *T. ovis*, *T. sergenti/T. buffeli/T. orientalis*, *T. annulata*, and *T. sinensis*) were detected using genus- and species-specific primers. It has been speculated that in areas with high infection rates, there is an inverse relationship between age and resistance to infection, where fawns gradually acquire immunity without showing clinical symptoms, and immunity is maintained by repeated challenges with the parasites. Consequently, a persistent parasite reservoir is established in the wild ruminants [16]. However, in these hosts, stressors like high parasitemia, poor nutrition, high population density, harsh weather conditions, or handling (e.g. translocation) can lead to symptomatic piroplasmosis, a cause of severe disease and death [15].

The diagnosis of parasitic diseases and detection of parasite species is often difficult at the carrier stage and in animals with mixed infections. Nevertheless, molecular diagnostic methods can enable the direct, specific, and sensitive detection of parasite species [27,34]. PCR-based molecular techniques allow the detection and discrimination of these parasites at low parasitemias and in animals with mixed infections [33,34]. The PCR methods used in this survey can detect cervine *Theileria* and *Babesia* directly in the same samples based on the 18S rRNA gene sequence using *Theileria* and *Babesia* specific primer sets. In the present study, the prevalence of *Theileria* infections in red deer and silk deer, which were detected by PCR, was statistically higher than that observed via microscopic examination. These results were expected because carrier animals often have low parasitemias, which are difficult to detect microscopically.

The maximum parsimony tree of the 18S rRNA genes indicated that the cervid infections detected in this study could be divided into two groups within the *Theileria* species clade. Group 1 sequences fell within the low-pathogenicity clade containing *T. capreoli* BAB1158 (AY7266011: isolated from European roe deer in Spain), *Theileria* spp. 3185/02 (AY421708: isolated from red deer in Spain), and *Theileria* spp. 3185/02 (DQ866842: isolated from roe deer from the Basque Country of Spain), and were also present in roe deer and red deer from northern Spain [16], and in fallow deer from Italy [13], but absent from chamois. In contrast, group 2 *Theileria* spp. fell into the same clade as the high-pathogenicity *T. uilenbergi* Longde isolate (AY262120), as did the *T. uilenbergi* Lintan isolate (AY262116) and the *T. uilenbergi* Yongjing isolate (JF719835), which are all widely present in sheep and goats and transmitted by *Haemaphysalis qinghaiensis* and *H. longicornis* in northern and northwestern China; their sequences were almost identical to each other with high bootstrap values (Figure 1). Clustering of the 18S rRNA sequences into two groups indicates the heterogeneity of the 18S rRNA genes of cervine *Theileria* spp. in China. Therefore, the two *Theileria* spp. newly identified in Cervidae in northwestern China should be classified as *T. capreoli* and *T. uilenbergi*. The 18S rRNA gene sequence of *T. capreoli* was most closely related to *Theileria* CYN3B of Japan and *T. ovis*, which belong to an evolutionary distinct lineage of non-lymphoproliferative *Theileria* species. This species was clearly divergent from *Babesia* and a second lineage of lymphoproliferative *Theileria* species that included *T. annulata* and *T. parva.*

Ideally, tick transmission studies on ticks that are associated with infected herds should be conducted to identify the species involved in transmitting theileriosis to Cervidae. In this study, no ticks were collected from the deer or the surrounding environments of the two areas investigated because the sampling was carried out in August, a time of year when the ticks are inactive. Yin *et al.*[35] and Li *et al.*[36] identified *H. qinghaiensis* and *H. longicornis* as vectors for *T. uilenbergi* in China. *H. qinghaiensis* is a triphasic tick common in Northwestern China and *H. longicornis* is a ubiquitous triphasic tick found in most parts of China. Therefore, it can be speculated that *H. qinghaiensis* and *H. longicornis* may play an important role as natural vectors of *T. uilenbergi* in northwestern China. Galuppi *et al*. [13] detected *T. capreoli* DNA in *Ixodes ricinus*, but whether other vectors transmit *T. capreoli* remains unclear. At present, identification of the full range of tick species that can transmit *T. capreoli* to Cervidae awaits further investigation.

## Conclusions

Our results provide important data that should increase understanding of the epidemiology of cervine theileriosis, and assist with the implementation of measures to control theileriosis transmission to Cervidae and small ruminants in northwestern China. Clarification of the role that Cervidae might have as reservoir hosts for maintaining *T. capreoli* and *T. uilenbergi* is critical to understanding whether Cervidae contribute to the spread of ruminant theileriosis in China.

## Competing interests

The authors declare that they have no competing interests.

## Authors’ contributions

YL, ZC, ZL, and JL carried out the molecular genetic studies; JY, QL, YL; SC participated in the sequence alignment, GG and QR collected the samples; YL, JL and HY drafted the manuscript. All authors read and approved the final manuscript.
